# Longer-Term Effects of Cardiac Telerehabilitation on Patients With Coronary Artery Disease: Systematic Review and Meta-Analysis

**DOI:** 10.2196/46359

**Published:** 2023-07-28

**Authors:** Wen Zhong, Rui Liu, Hongxin Cheng, Lin Xu, Lu Wang, Chengqi He, Quan Wei

**Affiliations:** 1 Department of Rehabilitation Medicine and Institute of Rehabilitation Medicine West China Hospital Sichuan University Chengdu, Sichuan China; 2 Key Laboratory of Rehabilitation Medicine in Sichuan Province Chengdu, Sichuan China

**Keywords:** cardiac telerehabilitation, coronary artery disease, CAD, cardiac rehabilitation, CR, long-term effect, meta-analysis

## Abstract

**Background:**

Cardiac telerehabilitation offers a flexible and accessible model for patients with coronary artery disease (CAD), effectively transforming the traditional cardiac rehabilitation (CR) approach.

**Objective:**

This systematic review and meta-analysis aimed to evaluate the long-term effectiveness of cardiac telerehabilitation.

**Methods:**

We searched randomized controlled trials (RCTs) in 7 electronic databases: PubMed, Web of Science, EMBASE, the Cochrane Central Register of Controlled Trials, ClinicalTrials.gov, the China National Knowledge Infrastructure, and WANFANG. The primary outcome focused on cardiopulmonary fitness. For secondary outcomes, we examined cardiovascular risk factors (blood pressure, BMI, and serum lipids), psychological scales of depression and anxiety, quality of life (QoL), cardiac telerehabilitation adherence, and adverse events.

**Results:**

In total, 10 RCTs fulfilled the predefined criteria, which were reviewed in our meta-analysis. The results showed that after cardiac telerehabilitation, there was a significant difference in the improvement in long-term peak oxygen uptake compared to center-based CR (mean difference [MD] 1.61, 95% CI 0.38-2.85, *P*=.01), particularly after 6-month rehabilitation training (MD 1.87, 95% CI 0.34-3.39, *P*=.02). The pooled effect size of the meta-analysis indicated that there were no significant differences in the reduction in cardiovascular risk factor control. There was also no practical demonstration of anxiety scores or depression scores. However, cardiac telerehabilitation demonstrated an improvement in the long-term QoL of patients (MD 0.92, 95% CI 0.06-1.78, *P*=.04). In addition, the study reported a high completion rate (80%) for cardiac telerehabilitation interventions. The incidence of adverse events was also low during long-term follow-up.

**Conclusions:**

Cardiac telerehabilitation proves to be more effective in improving cardiopulmonary fitness and QoL during the long-term follow-up for patients with CAD. Our study highlights monitoring-enabled and patient-centered telerehabilitation programs, which play a vital role in the recovery and development of CAD and in the long-term prognosis of patients.

## Introduction

Cardiovascular diseases (CVDs) are the leading cause of a large proportion of deaths worldwide, accounting for approximately 1/3 of all deaths [1]. The global burden of CVDs is rising, increasing from 271 million in 1990 to 523 million in 2019, and the total number of cases of CVDs have almost doubled, especially coronary artery disease (CAD) [2]. CAD occurs mainly due to the progression of atherosclerosis of coronary arteries to narrowing or occlusion, leading to blood flow limitation, which causes cardiomyocyte or myocardial necrosis [3]. Population growth and aging are the main drivers, and the prevention and control of CVDs face significant challenges.

Cardiac rehabilitation (CR) is a complex, multidisciplinary intervention aimed at comprehensive rehabilitation assessment of the patient’s condition to meet the needs of patients with CVD, preventing disease recurrence and further progression. CR involves various components, such as exercise training, drug counseling, diet, nutrition, psychological regulation, and risk factor management [4]. To better serve patients with CAD, this multidisciplinary treatment method can be combined with well-validated strategies to assess physical function and risk factors in order to personalize treatment strategies [5]. Studies have shown that CR can reduce the risk of recurrent heart attacks by 47%, heart disease mortality by 36%, and all-cause mortality by 26% [6]. Furthermore, secondary prevention recommendations emphasize CR’s importance in patients with CAD, which has been recognized as comprehensive medical monitoring to reduce CAD mortality, morbidity, disability, and high expense [7,8]. Although participation in CR is a class IA recommendation for CAD [9], rates of referral and usage remain low [8]. Aragam et al [10] demonstrated that approximately >40% of patients are not referred for CR after percutaneous coronary intervention (PCI) by the time of hospital discharge. Participation enrollment in CR ranges from only 20% to 30% in the United States [8]. Low participation and adherence to CR programs may be attributed to multifactorial conditions, such as comorbidities, living farther from medical organizations, high medical costs, and time commitment [11,12].

To alleviate these barriers and improve the uptake rates of CR, cardiac telerehabilitation, a targeted approach, is used to effectively shift the traditional rehabilitation mode to a high-value overall strategy. Telerehabilitation is defined as a telemedicine platform, including telediagnosis, teletreatment, and remote monitoring [13]. These technologies are conducive to the joint participation of doctors and patients and medical departments in patients’ health management work. For patients with CVD, doctors can monitor the patients’ vital signs and cardiac telerehabilitation progress through a remote monitoring system and adjust the CR treatment plan according to the patients’ condition. Cardiac telerehabilitation uses internet information technology to allow patients to receive rehabilitation treatment at home or in other nonhospital settings regardless of time and geographical restrictions. This can motivate and help supervise patients, improving patient compliance. These factors have promoted the popularity of cardiac telerehabilitation, and the comprehensive promotion of telemedicine construction and development is gradually focusing on this aspect. Most patients eligible for cardiac telerehabilitation have a low rate of adverse events during exercise training if previously adequately evaluated. A review by Stefanakis et al [14], which included 5 studies on adverse event rates in home telerehabilitation, estimated the incidence of adverse events in the sample to be 1 in 23,823 patient-hours of exercise.

As a more accessible and flexible model of CR, cardiac telerehabilitation has been developed based on new communication technologies and advanced telemedical devices, such as smartphones, web-based apps, wearable sensors, and virtual reality [15]. This is supported by recent meta-analyses [16,17] that have shown that cardiac telerehabilitation as an alternative rehabilitation delivery model achieves an equivalent effect on physical exercise capacity, behavior change, reduction in risk factors, and improvement in the quality of life (QoL) of patients with CAD compared to the traditional rehabilitation model. At present, in addition to the traditional outpatient rehabilitation model, the main approaches to the CR model include home-based CR and cardiac telerehabilitation. Both cardiac telerehabilitation and home-based CR refer to rehabilitation in a nonhospital setting and have their advantages. Telerehabilitation is delivered and implemented through telemedical equipment, while home-based CR refers to rehabilitation carried out in the patient’s home. Both approaches can serve as important supplements or alternatives to traditional in-hospital rehabilitation models. However, the main difference between the 2 approaches is that home-based CR patients rely on outpatient or community follow-up guidance, take subjective initiatives at home for rehabilitation, and lack uninterrupted supervision and guidance from doctors; cardiac telerehabilitation makes up for these disadvantages of home-based CR. Therefore, the control of patients participating in telerehabilitation is strict. In a 2019 joint statement, the American Association of Cardiovascular and Pulmonary Rehabilitation (AACVPR), the American Heart Association (AHA), and the American College of Cardiology (ACC) [5] suggested that cardiac telerehabilitation could be a reasonable alternative for patients with clinically stable, low-to-moderate-risk CVD. However, high-risk patients, such as patients with unstable angina, CVD with heart failure or symptomatic arrhythmias, and other hemodynamic instabilities, usually require careful evaluation, outpatient CR under supervision, and reassessment for stabilization during the convalescent phase before participating in cardiac telerehabilitation.

Cardiac telerehabilitation has been found to be effective, as evidenced by improvements in the condition of patients with CVD. Still, current telerehabilitation studies have aimed to assess whether telerehabilitation affects short-term (about 3-month follow-up) or medium-term (about 6-month follow-up) effectiveness [18-20]. There are limited data describing the long-term (more than 1-year follow-up) effects of telerehabilitation. A self-regulation lifestyle program [21] reported that motivation for lifestyle changes tends to diminish. At the same time, patients with CAD feel healed, which influences long-term beneficial changes in lifestyle and risk factors. Hence, evaluating the long-term effectiveness of telerehabilitation has practical significance for implementing CR. Considering the well-established association between telerehabilitation and the potential beneficial effects in CAD, in this study, we hypothesized that cardiac telerehabilitation could maintain the results for longer-term consequences.

## Methods

### Study Design

This meta-analysis was performed according to the Preferred Reporting Items for Systematic Review and Meta-Analyses (PRISMA) statement.

### Ethical Considerations

All analyses were based on previously published studies, so no ethical approval or patient consent was needed.

### Literature Search

A literature search was performed to identify relevant studies in the following 7 electronic databases: PubMed, Web of Science, EMBASE, the Cochrane Central Register of Controlled Trials (CENTRAL), ClinicalTrials.gov, the China National Knowledge Infrastructure (CNKI), and WANFANG.

The search proceedings used the following different keywords without a time or language limit: (coronary artery disease or left main coronary artery disease or coronary arteriosclerosis) AND (telerehabilitation or tele-rehabilitation or tele rehabilitation or remote rehabilitation or virtual rehabilitation or telemedicine or mobile health or telehealth or eHealth or internet or online or web or sensor or wearable or smartphone or App or WeChat or QQ). Multimedia Appendix 1 provides the complete search strategy. To retrieve a comprehensive list of eligible papers, we manually screened reference lists, relevant conference lists, and even gray literature.

### Inclusion and Exclusion Criteria

We defined and applied explicit inclusion criteria to select eligible studies for this meta-analysis as follows:

- Population: adults (≥18 years old) diagnosed with CVD (stable angina pectoris, acute coronary syndrome, myocardial infarction, or postcoronary revascularization, such as PCI or coronary artery bypass grafting [CABG]).
- Intervention: We focused on telerehabilitation as an emerging model of rehabilitation service delivery based on smartphones, wearable monitoring portable devices, virtual reality, or other internet interventions. Modern telecommunications technology combined with rehabilitation is designed to complete functional exercise training sessions, achieve self-management, and improve physical function. Patients are available at home with remote monitoring and remote health care consultation.
- Comparison: A control group was randomly assigned to usual-care or center-based CR.
- Outcomes: The primary outcome focused on cardiopulmonary function assessed with the peak oxygen uptake (peak VO_2_) using the cardiopulmonary exercise test (CPET). For secondary outcomes, we focused on changes in cardiovascular risk factors, psychological scales of depression and anxiety, QoL, cardiac telerehabilitation adherence, and adverse events.
- Study design: Randomized controlled trials (RCTs) that compared the cardiac telerehabilitation group with the control group and evaluated the longer-term effects during least 12 months’ follow-up were included in the review.

We also set the following exclusion criteria: (1) patients with severe heart failure with New York Heart Association (NYHA) functional class III or IV, malignant cardiomyopathy, valvular disease, heart failure; (2) papers with an unreasonable literature research design, non-RCTs, nonhuman or animal studies, or a follow-up time of <12 months; (3) repeated publication; (4) unavailable full text, incomplete information and data, or an inability to extract and compare data; and (5) conference papers, abstracts, reviews, letters to the editor, and case reports.

### Selection of Studies

Potentially relevant papers meeting the abovementioned search strategy were imported into the EndNote X9.2 tool (Clarivate). Initially, 2 reviewers each independently screened the titles and abstracts of all studies on the finalized list. Next, they conducted full-text screening according to the inclusion and exclusion criteria to determine the final eligibility. During the overall flow of the process, if there were any different views, a third reviewer provided an opinion and resolved the disagreement via consensus.

### Data Extraction

Two authors collaborated on the final decision of data extraction, which was summarized in Microsoft Word 2019 and Microsoft Excel 2019: (1) study design (eg, first author, year of publication, country, study design, follow-up time), (2) participants (eg, sample size, sex, age, diagnosis), (3) intervention (eg, telerehabilitation group vs control group), (4) change in our protocol-specified outcomes, and (5) risk of bias. Although relevant details were insufficiently reported in the included studies, we contacted the authors via email for further information.

### Risk-of-Bias and Quality Assessment of Studies

We evaluated each study’s eligibility using the Cochrane risk-of-bias tool [22] to assess the risk of all types of bias (selection bias, performance bias, attrition bias, reporting bias, and other sources of bias). Furthermore, we also used the Physiotherapy Evidence Database (PEDro) scale [23] to perform a quality assessment of the studies included. The PEDro scale comprises 11 items that correspond to a maximum of 10 points, except for item 1. A study with a PEDro score of 9-10 points is considered excellent, a score of 6-8 points is considered good, and a score of ≤5 is considered poor (low level of quality). The quality of the studies was independently assessed by 2 authors, and any dissent was settled through discussion or via consultation with a third reviewer.

### Statistical Analysis

Data analysis and synthesis were performed using Cochrane Review Manager (RevMan) version 5.2 for Windows. Although all RCTs follow the principle of randomization and most baseline characteristics have no significant difference, we calculated the change from initial to final follow-up treatment difference values with a correlation coefficient of 0.5 [24] to obtain a more accurate comparison of changes in the outcomes. For continuous data, we presented the outcomes using the mean difference (MD) with 95% CIs, and *P*<.05 was considered statistically significant. If studies used different units or measurement scales, we used the standardized mean difference (SMD) with 95% CIs. As a basis for assessing heterogeneity, an inconsistency of I^2^ test values of more than 50% was considered indicative of substantially high heterogeneity. If we observed statistical heterogeneity with a threshold of >50%, random-effect models were used; otherwise, fixed-effect models were applied. In addition, if this threshold was exceeded, we performed a leave-one-out sensitivity analysis to ascertain whether our findings were driven by a single study, and checked the potential reasons for heterogeneity.

## Results

### Search and Study Selection

**Figure 1:**
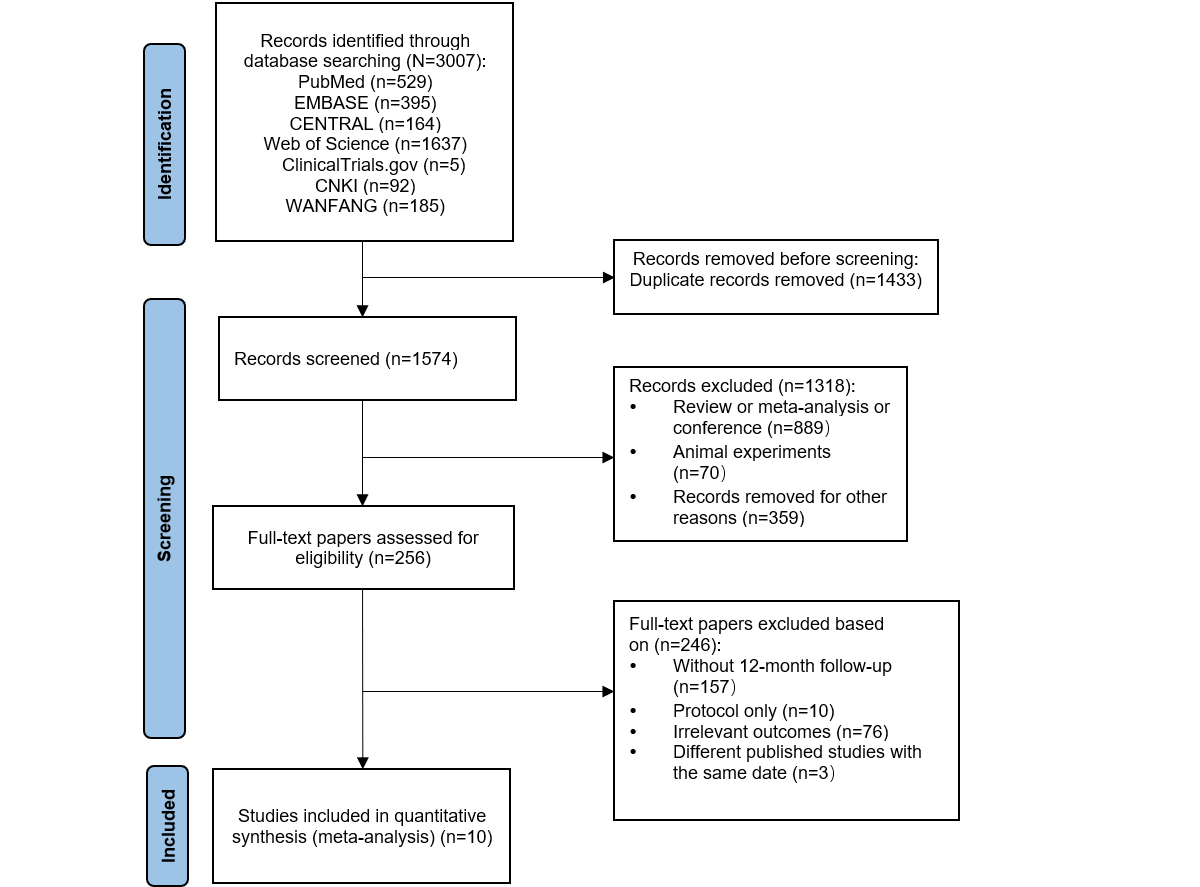
The Preferred Reporting Items for Systematic Reviews and Meta-Analyses (PRISMA) flow diagram for the selection of studies. CENTRAL: Central Register of Controlled Trials; CNKI: China National Knowledge Infrastructure.

A comprehensive overview of the study screening and selection process is presented using a PRISMA 2020 flow diagram in Figure 1. The diagram provides a visual representation of the selection process, while the exclusion and inclusion reasons are explained in detail later. A total of 3007 citations were identified, and 1433 (47.7%) duplicates were excluded. Of the remaining 1574 (52.3%) papers that underwent title and abstract screening, 256 (16.3%) were found to be potentially relevant to the research topic. For further assessment, full-text screening was performed, and 10 (3.9%) RCTs fulfilled the predefined criteria and were incorporated into our systematic review and meta-analysis. Importantly, a significant number of intervention-related RCTs were excluded from the analysis due to having a follow-up period of less than 12 months.

### Study Characteristics

Descriptive characteristics of the 10 studies [25-34] were captured and are summarized in Multimedia Appendix 2, including study design, main follow-up time, population, intervention, control setting, and measured outcomes. Of these 10 studies, 9 (90%) [26-34] were designed as 2-arm prospective RCTs with a parallel design, and the remaining study [25] was a 3-arm RCT. In addition, 2 (20%) studies each were performed in the Netherlands [30,33], China [28,34], Canada [31,32], and Belgium [25,29] and 1 (10%) each in the Czech Republic [26] and Spain [27]. In total, 1417 participants completed the RCTs, with 709 (50%; n=123, 17.3%, women and n=586, 82.7%, men) participants in the intervention group and 708 (50%; n=119, 16.8%, women and n=589, 83.2%, men) participants in the control group. The mean age of the intervention and control groups at baseline was 59.1 (SD 9.5) years and 59.9 (SD 9.8) years, respectively (*P*=.16).

### Intervention Programs

In our systematic review, CR involved multiple components, mainly focusing on exercise intervention, risk factor management, medical evaluation, reasonable dietary combinations, and psychosocial counseling [35]. In the papers included, these telemonitoring and telerehabilitation-delivered CR interventions were implemented using different remote application equipment, as summarized in Multimedia Appendix 2. Depending on how a network (the internet) is accessed and the devices used, there are different design options available for the presentation of cardiac telerehabilitation. For example, Kraal et al [30] and Snoek et al [33] used wearable monitoring devices to enable real-time monitoring of patients’ personalized exercise training. Avila et al [25], Batalik et al [26], Frederix et al [29], Kraal et al [30], and Snoek et al [33] focused mainly on exercise-based cardiac telerehabilitation, with a predominantly moderate exercise intensity, a training heart rate equivalent to 70%-80% of the heart rate reserve, and individualized control through electronic monitoring. Among them, only Snoek et al [33] used an exercise intensity not only above the first ventilation threshold but also in the moderate exercise intensity range, approximately equal to 70%-80% of the heart rate reserve. When constructing personalized exercise sessions, the purpose of exercise also needs to be considered, and different exercise methods can have different health benefits. We included studies that mainly aimed to enhance cardiorespiratory fitness, choosing walking, jogging, and cycling, while Blasco et al [27], Reid et al [32], and Wang et al [34] performed some aerobic exercises in a home environment according to their preferences. However, Batalik et al [26], Kraal et al [30], Snoek et al [33], and particularly Frederix et al [29] and Dorje et al [28] recorded patients’ walking to assess their exercise condition. In addition to enhancing cardiorespiratory function, it is also vitally essential to increase muscle strength and endurance, and most of the exercises involved in these studies are aerobic exercises. Only Avila et al [25] used strength exercises, such as arm ergometry and rowing, but did not report whether strength training, such as weightlifting, sit-ups, and push-ups, was used. However, there was a lack of exercise to improve flexibility and body coordination. In 5 (50%) studies, Blasco et al [27], Dorje et al [28], Lear et al [31], Reid et al [32], and Wang et al [34], the focus was on structured comprehensive CR, including risk factor management for CVD, emotional management, dietary management, and medication management, and the studies also involved the core component of exercise coaching. Although these studies did not have detailed exercise prescriptions, using social platforms, such as WeChat, to provide more comprehensive CR, with motivational feedback about progress, can lead to better effects.

Moreover, Avila et al [25], Frederix et al [29], and Reid et al [32] supervised home exercises and uploaded web-based reports to motivate patients to improve adherence and self-management enthusiasm. Batalik et al [26], Blasco et al [27], Dorje et al [28], Lear et al [31], and Wang et al [34], mainly used educational videos or electronic pamphlets supported by WeChat and other apps, which enabled medical staff to communicate online with patients. Patients could carry out remote health consultations to improve their QoL and control cardiac risk factors.

### Risk of Bias

**Figure 2:**
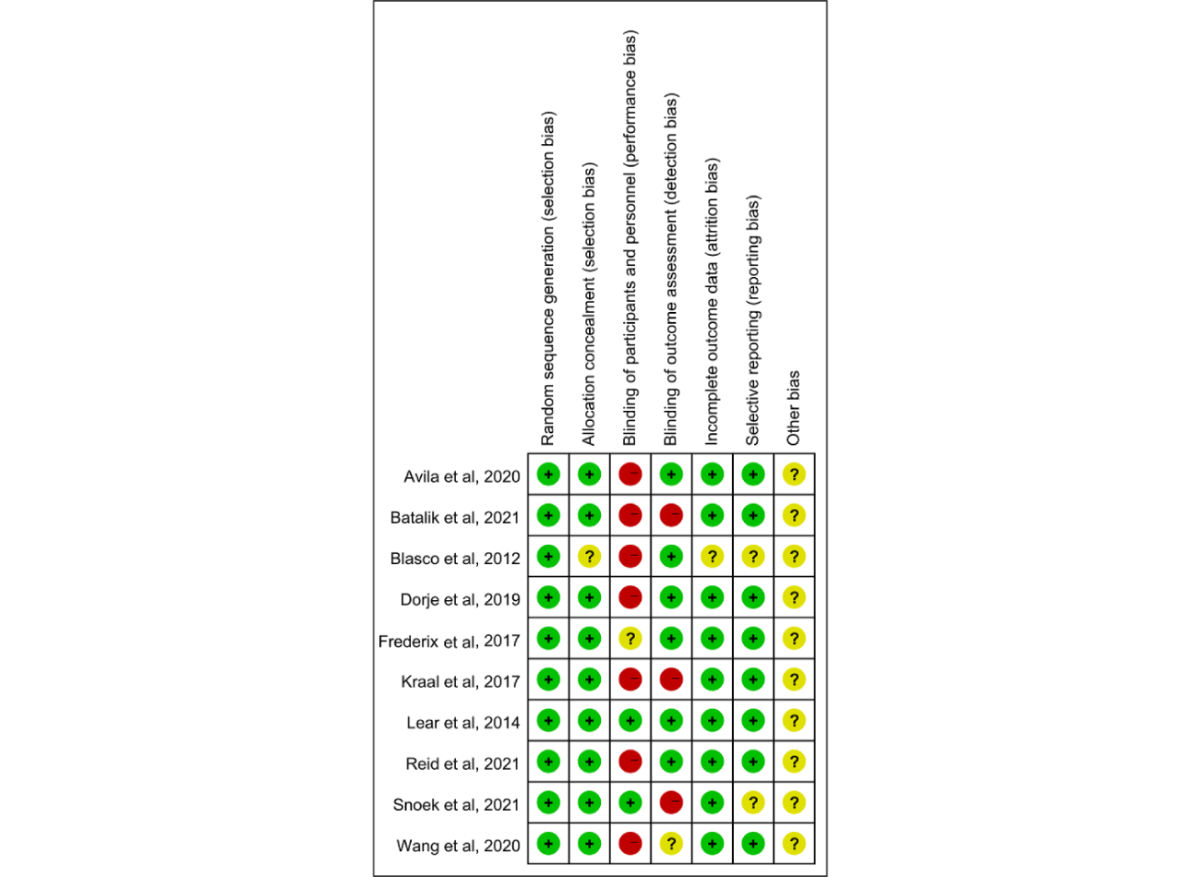
Risk-of-bias summary: the authors’ judgments about each risk-of-bias item for each included study were reviewed. Red, green, and yellow colors indicate high, low, and unclear risk of bias, respectively.

All the 10 (100%) studies analyzed in this review using the PEDro scale had acceptable methodological quality (score≥6); see Table 1. The results of the risk-of-bias assessment for the included studies are graphically displayed in Figure 2. We first used the Cochrane risk-of-bias tool, including selection, performance, attrition, and reporting biases. The 10 studies described specific randomization methods, 9 (90%) [25,26,28-34] reported allocation concealment, while 1 (10%) [27] had no allocation concealment. All studies had no subject blinding, and most studies had no therapist blinding due to regular supervision and timely feedback in the rehabilitation environments. Moreover, 8 (80%) studies had no reporting bias, 2 (20%) were unclear, and all studies had no clear descriptions of other biases.

**Table 1:** The PEDro^a^ scale to assess the included RCTs’^b^ methodological quality.

Quality metric	Author									
	Avila et al [25]	Batalik et al [26]	Blasco et al [27]	Dorje et al [28]	Frederix et al [29]	Kraal et al [30]	Lear et al [31]	Reid et al [32]	Snoek et al [33]	Wang et al [34]
Eligibility criteria^c^	1^d^	1	1	1	1	1	1	1	1	1
Random allocation	1	1	1	1	1	1	1	1	1	1
Concealed allocation	1	1	0^e^	1	1	1	1	1	1	1
Baseline comparability	1	1	1	1	1	1	1	1	1	1
Blinded subjects	0	0	0	0	0	0	0	0	0	0
Blinded therapists	0	0	0	0	0	0	1	1	1	0
Blinded assessors	0	0	1	1	1	0	1	1	0	0
Adequate follow-up	1	1	1	1	1	1	1	1	1	1
Intention-to-treat analysis	0	0	0	1	1	0	0	0	1	1
Between-group comparisons	1	1	1	1	1	1	1	1	1	1
Point estimates and variability	1	1	1	1	1	1	1	1	1	1
Total score	6	6	6	8	8	6	8	8	8	7

^a^PEDro: Physiotherapy Evidence Database.

^b^RCT: randomized controlled trial.

^c^Eligibility criteria did not contribute to the total score.

^d^1=yes (reported in the study).

^e^0=no (not met).

### Assessment of Outcomes

#### Cardiorespiratory Fitness

**Figure 3:**
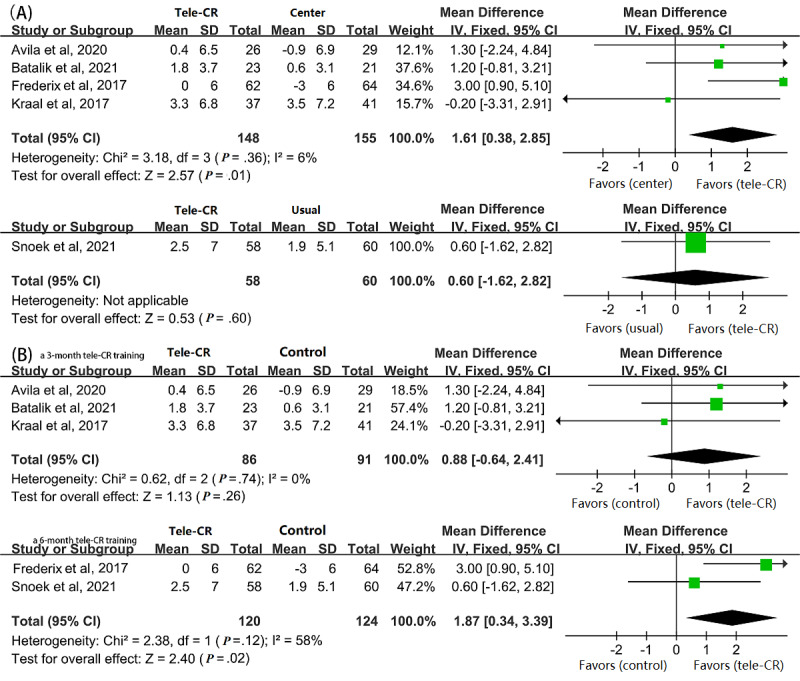
Pooled MD between cardiac telerehabilitation and control groups in terms of peak VO2 in long-term follow-up, divided into (A) cardiac telerehabilitation vs center-based CR or usual care and (B) a 3- or 6-month telerehabilitation program in the intervention group. CR: cardiac rehabilitation; MD: mean difference; peak VO2: peak oxygen uptake.

We included 10 studies on people with CVD that evaluated long-impact cardiac telerehabilitation interventions, 5 (50%) [25,26,29,30,33] of the 10 RCTs reported peak VO_2_, and a total of 421 participants who had at least 12 months of follow-up were included in the analysis. To exclude the effect of the type of control group intervention, stratified based on center-based CR or usual care, on outcomes, subgroup analyses of the 5 studies were performed, of which the control group of only 1 (20%) study, by Snoke et al [33], received usual care. Therefore, the combined meta-results of the analyses of the remaining 4 (80%) studies showed that real-time monitored exercise-based cardiac telerehabilitation significantly improves long-term peak VO_2_ compared to center-based CR (MD 1.61, 95% CI 0.38-2.85, *P*=.01), as shown in Figure 3A. Subgroup analyses were also performed for patients considering the different effects of intervention durations in exercise protocols. As shown in Figure 3B, peak VO_2_ improvement in the intervention group was significantly greater than that in the control group after 6-month telerehabilitation training (MD 1.87, 95% CI 0.34-3.39, *P*=.02), but there was no significant difference after 3-month telerehabilitation training.

#### Cardiovascular Risk Factors

**Figure 4:**
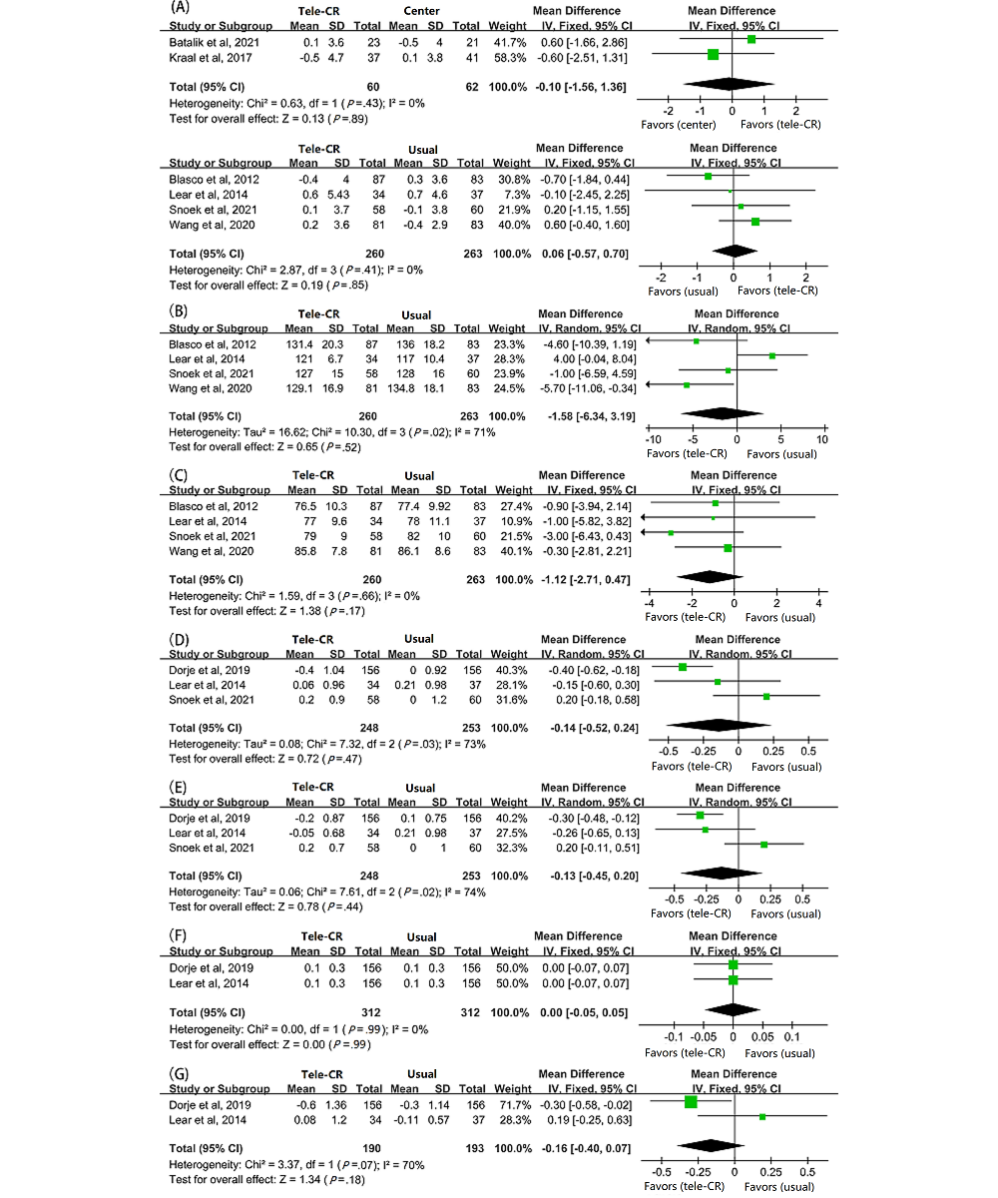
Pooled MD between the cardiac telerehabilitation and control groups in terms of (A) BMI, (B) systolic blood pressure, (C) diastolic blood pressure, (D) total cholesterol (TC), (E) low-density lipoprotein cholesterol (LDL-C), (F) high-density lipoprotein cholesterol (HDL-C), and (G) triglycerides (TGs) in long-term follow-up. CR: cardiac rehabilitation; MD: mean difference.

In our study, cardiovascular risk factors mainly included the BMI, blood pressure, and lipid profile. Of the 10 studies, 6 (60%) [26,27,30,31,33,34] reported the BMI after long-term follow-up, showing no significant difference between cardiac telerehabilitation and center-based CR (MD –0.10, 95% CI –1.56 to 1.36, *P*=.89; I^2^=0%) or between cardiac telerehabilitation and usual care (MD –0.60, 95% CI –0.57 to 0.70, *P*=.85; I^2^=0%); see Figure 4A. In terms of blood pressure, there was no significant difference in systolic blood pressure (MD –1.58, 95% CI –6.34 to 3.19, *P*=0.52; I^2^=0%; Figure 4B) and diastolic blood pressure (MD –1.12, 95% CI –2.71 to 0.47, *P*=0.17; I^2^=0%; Figure 4C) compared to the usual-care combined effect size. Only 4 (40%) studies [28,29,31,33] included the blood lipid index as an outcome measure, of which 3 (75%) studies [28,31,33] used usual care for the control group, and only Frederix et al [29] used center-based CR. Therefore, to maintain the consistency of the control group and reduce bias, the 3 (75%) studies [28,31,33] were finally included to analyze the improvement in blood lipids. The results showed that there was no more significance than the usual-care group in improving total cholesterol (TC; MD –0.14, 95% CI –0.52 to 0.24, *P*=.47; I^2^=73%; Figure 4D), low-density lipoprotein cholesterol (LDL-C; MD –0.13, 95% CI –0.45 to 0.20, *P*=.44; I^2^=74%; Figure 4E), high-density lipoprotein cholesterol (HDL-C; MD 0.00, 95% CI –0.05 to 0.05, *P*=.99; I^2^=0%; Figure 4F), and triglycerides (TGs; MD –0.16, 95% CI –0.40 to 0.07, *P*=.18; I^2^=70%; Figure 4G).

#### Depression and Anxiety

**Figure 5:**
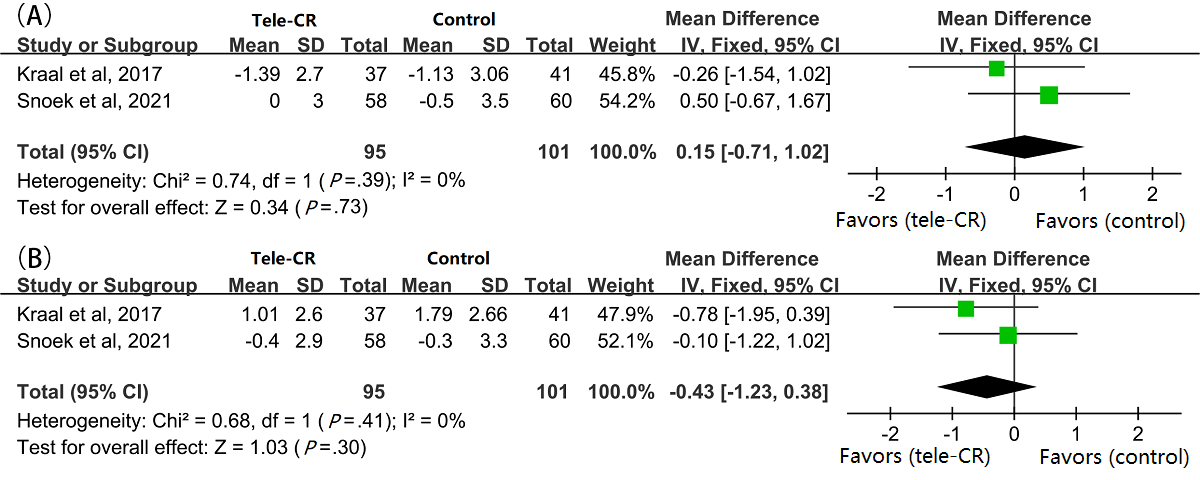
Pooled MD between the cardiac telerehabilitation and control groups in terms of (A) anxiety score change and (B) depression score change in long-term follow-up. CR: cardiac rehabilitation; MD: mean difference.

Of the 10 studies included, 2 (20%) [30,33] applied fixed-effect meta-analysis and showed no significant long-term improvement in anxiety scores (MD 0.15, 95% CI –0.71 to 1.02, *P*=.73; I^2^=0%; Figure 5A) or depression scores (MD –0.43, 95% CI –1.23 to 0.38, *P*=.30; I^2^=0%; Figure 5B).

#### Quality of Life

**Figure 6:**
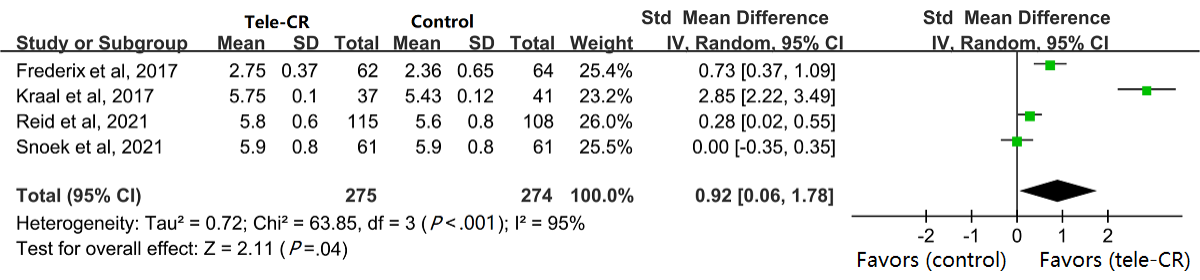
Pooled SMD in terms of QoL. CR: cardiac rehabilitation; QoL: quality of life; SMD: standardized mean difference.

Due to the different scales used to measure QoL, we used the SMD as a practical measure. The MacNew Heart Disease Health-related Quality of Life (MacNew) questionnaire was used to explore the effects of cardiac telerehabilitation on QoL in people with CVD in 3 (30%) of the 10 studies included [30,32,33], and 1 (10%) study [29] used the HeartQoL questionnaire. However, the combined results showed high heterogeneity (I^2^=95%; Figure 6), and I^2^ still fluctuated between 70% and 95% after sensitivity analysis, so the random-effect model was used for analysis. The results showed that cardiac telerehabilitation could improve the long-term QoL of patients with CVD (MD 0.92, 95% CI 0.06-1.78, *P*=.04).

#### Adherence to the Telerehabilitation Program

**Figure 7:**
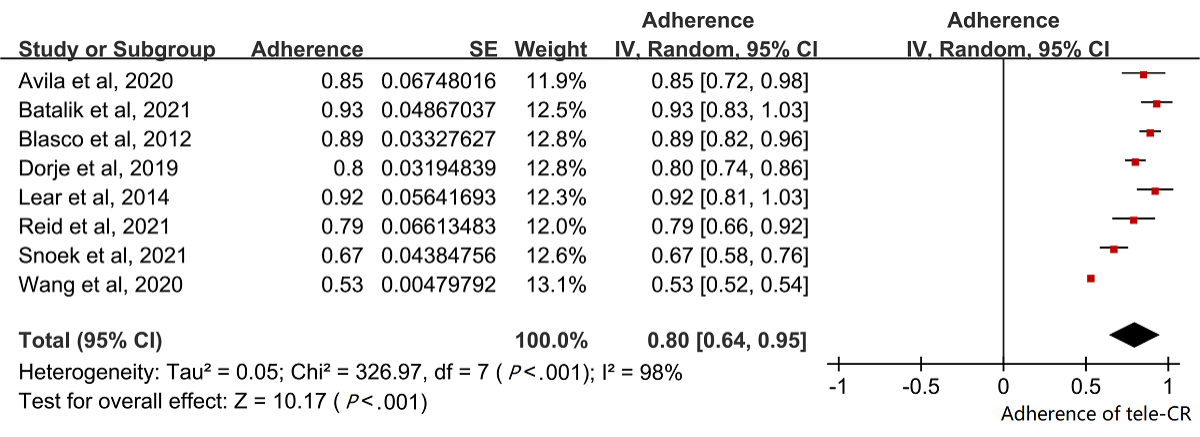
Pooled completion rate. CR: cardiac rehabilitation.

Completion rates for cardiac telerehabilitation were reported in 8 (80%) of the 10 studies (MD 0.80, 95% CI 0.64-0.95; Figure 7) [25-28,31-34], with high heterogeneity (I^2^=98%) based on our pooled meta-analysis.

#### Adverse Events

**Figure 8:**
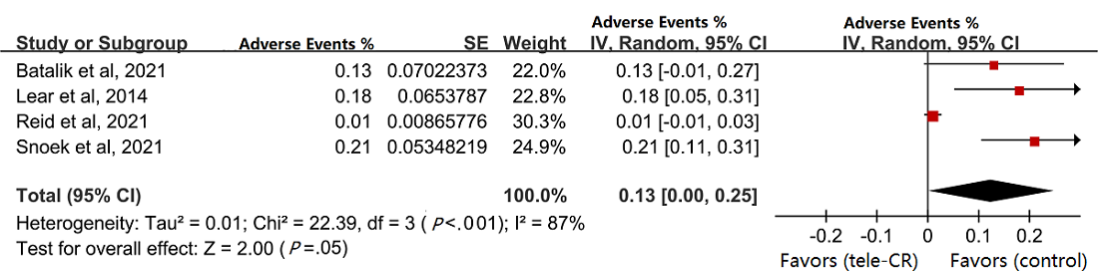
Pooled adverse event rate.

Of the 10 studies, in 4 (40%) [26,31-33], 13% of patients reported all-cause adverse events (MD 0.13, 95% CI 0.00-0.25; see Figure 8).

## Discussion

### Principal Findings

Compared to center-based CR, cardiac telerehabilitation was effective in improving cardiorespiratory fitness and exercise capacity in patients with CAD, particularly in terms of peak VO_2_, during long-term follow-up. However, technology-based cardiac telerehabilitation had no long-term benefits in terms of risk factor management. Based on the RCTs included, we also found that telehealth interventions do not result in significant improvements in depression and anxiety scores in the long term. Nevertheless, there was evidence of improved long-term QoL with cardiac telerehabilitation. Our study also revealed positive adherence to cardiac telerehabilitation interventions, and the incidence of adverse events during long-term follow-up was low.

Cardiorespiratory fitness is a crucial determinant of CR and a strong predictor of all-cause mortality and cardiovascular mortality [36]. The peak VO_2_ in CPET is 1 of the most critical and gold-standard indicators of cardiorespiratory fitness in patients with CVD, representing the maximum oxygen intake by the human body per unit body weight (mL/kg/minute). Peak VO_2_ reflects cardiopulmonary function in transporting oxygen and carbon dioxide around the body [37], the maximum aerobic metabolic capacity [38], and the skeletal muscles’ ability to absorb and use oxygen [39]. Our study confirms that compared to center-based CR training, cardiac telerehabilitation can significantly improve long-term peak VO_2_ in patients. Furthermore, we found that the duration of exercise training has a positive impact on the improvement in peak VO_2_. There is debate over whether ambulatory cardiac telerehabilitation is superior to traditional in-hospital or center-based CR, and this has also been widely discussed in recent years. Our primary finding, consistent with previous studies and related reviews [40-42], showed that exercise-based cardiac telerehabilitation with remote monitoring is equivalent or superior to center-based CR in terms of exercise capacity in cardiac disease. This can help patients overcome barriers such as transportation limitations and conflicts with work schedules, thereby expanding the implementation of rehabilitation programs for a wider range of patients. However, it is worth noting that these previous studies have primarily focused on short- or medium-term follow-up, and there is a lack of long-term evaluation. Therefore, our results further support the notion that compared to center-based CR, cardiac telerehabilitation can better sustain patients’ cardiorespiratory endurance over an extended period. Our finding is consistent with a retrospective study performed by Ramadi et al [43], who provided evidence that an extensive multidisciplinary and structured CR program retains the improvement in exercise capacity until 1-year follow-up. Interestingly, Aamot et al [44] and Smith et al [45] indicated that the monitoring strategy in CR enables the therapist to help patients sustain long-term exercise adherence so that peak VO_2_ significantly increases compared to baseline values. To strengthen our results, we also focused on other variables of CPET. Unexpectedly, we did not observe a significant difference. For example, only 2 RCTs [25,46] reported oxygen consumption at the anaerobic threshold (VO_2_ AT, mL/kg/minute) and showed that the improvement in VO_2_ AT does not persist for long. These indicators, especially VO_2_ AT, have a strong correlation with the clinical symptoms of patients with CVD [47], so further studies can evaluate whether cardiac telerehabilitation protocols can improve long-term clinical outcomes in order to strengthen the evidence that cardiac telerehabilitation can maintain cardiopulmonary fitness levels and exercise capacity.

Although our study and previous studies have shown that cardiac telerehabilitation shows significant long-term effectiveness in increasing peak VO_2_, the current intervention methods for cardiac telerehabilitation show considerable variability in their design. For example, in the 10 studies included in this review, Avila et al [25], Batalik et al [26], Kraal et al [30], and Snoek et al [33] used heart rate monitors, such as heart rate belts and sports bracelets, to detect exercise intensity and record health data in order to promptly synchronize patients’ exercise status. Blasco et al [27], Frederix et al [29], Lear et al [31], and Reid et al [32] used self-health management systems, such as smartphones or computers, to transmit patients’ blood sugar levels, blood pressure, smoking status, and other daily life conditions to the online platform to manage CVD risk factors; Frederix et al [29] also used activity trackers to monitor patients’ exercise. Furthermore, with continuous software and hardware optimization, Dorje et al [28] and Wang et al [34] used social software, such as the WeChat group mode, for education, management, and follow-up, as well as emotional and nutrition management. Although remote methods of intervention vary, at the core, they are the same; that is, using various types of remote equipment, they mobilize the subjective initiative of patients for rehabilitation, under the effective communication of medical care, for them to implement rehabilitation treatments, such as exercise prescription, drug prescription, psychological prescription, and risk factor management. Therefore, future remote CR content and the operation process will be more standardized.

Our subgroup analysis revealed that an extended intervention duration of 6 months in exercise protocols significantly improves and maintains cardiorespiratory fitness. However, compared to the control group, no significant difference was observed after the 3-month rehabilitation training. This could be attributed to the reinforcement of patient self-awareness of CR through extended telehealth guidance and monitoring. Slovinec et al [48] found that motivational orientation and self-efficacy of exercise behavior affect exercise maintenance and physical activity levels. Self-regulation theory [21] is supported by physical and mental aspects to motivate patients to realize their potential, actively face diseases and adverse reactions, and improve physical and mental health. Janssen et al [49] showed that a theory-based lifestyle program could stimulate and sustain improvements in exercise adherence. Therefore, cardiac telerehabilitation delivered through modern network technology has the potential to enhance long-term effectiveness by tailoring, coaching, monitoring, and providing objective feedback programs that enhance patients’ self-efficacy and subjective initiative. Maddison et al [50] indicated that mobile health interventions have a positive therapeutic effect on leisure-time physical activity and walking, which may be moderated by changes in self-efficacy, which also strengthens our conclusions.

We found no strongly favorable evidence of a difference in cardiovascular risk factor management. For cardiovascular risk factors, this meta-analysis indicated no significant differences in reduction in blood pressure, BMI, and blood lipid analysis changes over 12 months. Evaluation and management of risk factors are crucial for the prognosis of patients with CVD [5,51]. Furthermore, with the younger age of patients with CVD and the increase in human life expectancy worldwide, long-lasting beneficial changes are needed for targeted preventive activities, so future research projects in this field should focus on extending the efficacy of risk factor management and maintenance effects.

No effectiveness was demonstrated in anxiety and depression score changes compared to the control group. Negative emotions and the occurrence and development of CVDs are 2-way causes [52]. Penninx et al [53] carried out a 4-year follow-up of 2397 patients with undiagnosed CVD and found that patients with negative emotions are more likely to suffer from CVD than patients without mood disorders. Another study [54] found that CR can help people with anxiety and depression shift their attention, better vent their emotions, and effectively alleviate mood disorders. Internet-based cardiac telerehabilitation may enhance communication and feedback between patients and medical staff and achieve emotional problem solving on time [55]. There are few studies on telerehabilitation and emotions of patients with CVD. In addition to improving cardiopulmonary fitness and cardiovascular risk factor management, 1 of the ultimate goals of telerehabilitation is to improve the long-term QoL of people with CVD. There is a significant statistical difference between groups in long-term follow-up results. However, the effect of cardiac telerehabilitation on the QoL of patients with CVD may be influenced by different assessment tools, and there is no consensus at present. The relevant pathogenesis and treatment guidelines are imperfect, so further research is needed.

We found high participation rates in CR during long-term security follow-up in our study. Compliance with rehabilitation training is a key factor in improving the rehabilitation effect [56], due to the patients’ need to go to the hospital regularly for rehabilitation training, resulting in a poor participation rate and compliance, and medium- to long-term recovery rates after discharge are low [57]. Telerehabilitation compliance is high, which effectively improves the efficiency of the CR of patients. Ivers et al [58] showed that large multicenter RCT telehealth interventions can improve the completion rate of CR in patients with myocardial infarction, albeit only using simple and convenient remote methods, such as Short Messaging Service (SMS) and email, which was also verified in the summary results of the review by Santiago et al [59]. At the same time, cardiac telerehabilitation has a low incidence of adverse events if it is fully evaluated before implementation. For example, Piotrowicz et al [60] found no significant difference in the incidence of adverse events between 2 groups (12.5% vs 12.4%) during the 12- to 24-month follow-up after 9 weeks of cardiac telerehabilitation intervention in patients with heart failure compared to usual care. The most effective way to improve the safety of telerehabilitation is to fully assess the patient’s status, such as using CPET, noninvasive cardiac output, physical assessment, etc.

CR is a vital part of the rehabilitation process of patients with CVD. Based on the development and application of the internet and tele-equipment, cardiac telerehabilitation, as a new means of rehabilitation, can effectively carry out CR in the home-based environment, improve the participation and compliance of patients with CVD to undergo CR, and improve their functional status. Our study adds evidence to the advancement of telerehabilitation, in the hope that the popularization of telerehabilitation and the improvements in CR treatment for patients with CVD can improve the quality of rehabilitation, save medical time and medical costs, and solve the problem of some young patients being unable to participate in conventional center-based CR due to work. It is necessary for future studies to promote the “internet + cardiac rehabilitation” model to overcome the clinical problems of actual patients seeking medical treatment and effectively implement the model for every patient who needs CR.

### Limitations

There are some limitations of the study. First is the large variability and complexity of the interventions due to cardiac telerehabilitation delivering exercise intervention details according to the frequency, intensity, timing, and type (FITT) principle. Because relatively few trials are investigating the long-term outcomes of cardiac telerehabilitation, it is difficult to unify specifically detailed research protocols; this requires future investigations to verify the long-term effectiveness of cardiac telerehabilitation under different administration conditions and to develop a uniform personalized plan. Second, our study did not include as outcome measures long-term improvements in major adverse cardiovascular events (MACEs), all-cause mortality, or all-cause hospitalization in people with CVD. Cardiopulmonary fitness indicates that peak VO_2_ corresponds to a 13% reduction in all-cause mortality and a 15% reduction in cardiovascular mortality [61]. Therefore, future studies are required to include this indicator based on a sufficient sample size. Finally, we did not consider economic cost-effectiveness. Collecting data on patients’ medical cost burden and rehabilitation cycle during cardiac telerehabilitation and conducting financial analysis from a societal perspective are constructive. For example, Batalik et al [62] mentioned that cost-benefit analysis is essential for policy makers, systematic review of exercise-based telehealth CR is cost-effective, and the 12 studies included in their research showed no clear difference between telerehabilitation and center-based CR. Short- and long-term clinical-economic analyses are still required to facilitate the implementation of telerehabilitation interventions in the clinic.

### Conclusion

Cardiac telerehabilitation, as a promising treatment method, plays a crucial role in the comprehensive rehabilitation of patients with CVD. It addresses the diverse rehabilitation needs of patients and helps enhance their recovery. Our results demonstrated a significant difference in peak VO_2_ and QoL in terms of long-term improvements but no significant differences in changes in cardiovascular risk factor management and the psychological scales of depression and anxiety. Our results provide initial evidence supporting the use of cardiac telerehabilitation as an alternative model to center-based CR. By extending the benefits of cardiorespiratory effectiveness, cardiac telerehabilitation can promote patients’ long-term awareness of rehabilitation, thereby maximizing the prognosis for each patient.

## Appendix

Multimedia Appendix 1 Complete search strategy.

Multimedia Appendix 2 Descriptive characteristics of the 10 studies.

## Abbreviations

AT: anaerobic threshold
CAD: coronary artery disease
CPET: cardiopulmonary exercise test
CR: cardiac rehabilitation
CVD: cardiovascular disease
MD: mean difference
PCI: percutaneous coronary intervention
peak VO2: peak oxygen uptake
PEDro: Physiotherapy Evidence Database
PRISMA: Preferred Reporting Items for Systematic Review and Meta-Analyses
QoL: quality of life
RCT: randomized controlled trial
SMD: standardized mean difference
