# Supplementary material for: Longer-Term Effects of Cardiac Telerehabilitation on Patients With Coronary Artery Disease: Systematic Review and Meta-Analysis
Source: JMIR Mhealth Uhealth. 2023 Jul 28;11:e46359. doi: 10.2196/46359 (PMC10422170; doi:10.2196/46359)
Supplement: Multimedia Appendix 1 [file mhealth_v11i1e46359_app1.docx]

Search Strategy

Database: Pubmed

Date searched: 1 Dec 2022

Search Strategy:

--------------------------------------------------------------------------------

#1 " Coronary Artery Disease "[Mesh]

#2 ((((((((((((((Artery Disease, Coronary) OR (Artery Diseases, Coronary)) OR (Coronary Artery Diseases)) OR (Left Main Coronary Artery Disease)) OR (Left Main Disease)) OR (Left Main Diseases)) OR (Left Main Coronary Disease)) OR (Coronary Arteriosclerosis)) OR (Arterioscleroses, Coronary)) OR (Coronary Arterioscleroses)) OR (Atherosclerosis, Coronary)) OR (Atheroscleroses, Coronary)) OR (Coronary Atheroscleroses)) OR (Coronary Atherosclerosis)) OR (Arteriosclerosis, Coronary)

#3 #1 or #2

#4 "Telerehabilitation"[Mesh]

#5 (((((((((((Telerehabilitations) OR (Tele-rehabilitation)) OR (Tele rehabilitation)) OR (Tele-rehabilitations)) OR (Remote Rehabilitation)) OR (Rehabilitation, Remote)) OR (Rehabilitations, Remote)) OR (Remote Rehabilitations)) OR (Virtual Rehabilitation)) OR (Rehabilitation, Virtual)) OR (Rehabilitations, Virtual)) OR (Virtual Rehabilitations)

#6 " Telemedicine"[Mesh]

#7 ((((Mobile Health) OR (Health, Mobile)) OR (mHealth)) OR (Telehealth)) OR (eHealth)

#8 ((((((((Internet) OR (Web)) OR (Online)) OR (App)) OR (Wearable)) OR (Sensor)) OR (Smartphone)) OR (WeChat)) OR (QQ)

#9 #4 or #5 or #6 or #7 or #8

#10 randomized controlled trial[Publication Type] OR randomized[Title/Abstract] OR placebo[Title/Abstract]

#11 #3 and #9 and #10

Database: Embase

Date searched: 1 Dec 2022

Search Strategy:

--------------------------------------------------------------------------------

No. Query Results Results

#10. #1 AND #5 AND #9 395

#9. #6 OR #7 OR #8 794,355

#8. 'double-blind':ab,ti 214,456

#7. 'placebo':ab,ti 344,714

#6. 'random':ab,ti 392,585

#5. #2 OR #3 OR #4 771,839

#4. 'internet':ab,ti OR 'web':ab,ti OR 'online':ab,ti 722,600

OR 'app':ab,ti OR 'wearable':ab,ti OR

'sensor':ab,ti OR 'smartphone':ab,ti OR 'social

media':ab,ti OR 'wechat':ab,ti OR 'qqt':ab,ti

#3. 'telerehabilitation'/exp 1,906

#2. 'telemedicine'/exp 61,539

#1. 'coronary artery disease'/exp 384,969

Database: Cochrane Library

Date searched: 1 Dec 2022

Search Strategy:

--------------------------------------------------------------------------------

ID Search Hits

#1 MeSH descriptor: [Coronary Artery Disease] explode all trees 7207

#2 MeSH descriptor: [Telerehabilitation] explode all trees 176

#3 MeSH descriptor: [Telemedicine] explode all trees 3275

#4 (Internet):ti,ab,kw OR (Web):ti,ab,kw OR (Online):ti,ab,kw OR (App):ti,ab,kw OR (Wearable):ti,ab,kw (Word variations have been searched) 43937

#5 (Sensor):ti,ab,kw OR (smartphone):ti,ab,kw OR (WeChat):ti,ab,kw OR (QQ):ti,ab,kw (Word variations have been searched) 10584

#6 #2 or #3 or #4 or #5 52483

#7 #1 and #6 164

Database: Web of Science

Date searched: 1 Dec 2022

Search Strategy:

--------------------------------------------------------------------------------

1: TS=( Coronary Artery Disease* OR Artery Disease*, Coronary OR Left Main Coronary Artery Disease OR Left Main Disease* OR Left Main Diseases OR Left Main Coronary Disease OR Coronary Arteriosclerosis OR Arterioscleroses, Coronary OR Coronary Arterioscleroses OR Atherosclerosis, Coronary OR Atheroscleroses, Coronary OR Coronary Atheroscleroses OR Coronary Atherosclerosis OR Arteriosclerosis, Coronary)

2: TS=(Telerehabilitation* OR Tele-rehabilitation* OR Tele rehabilitation OR Remote Rehabilitation* OR Rehabilitation*, Remote OR Virtual Rehabilitation* OR Rehabilitation*, Virtual OR Telemedicine OR Mobile Health OR Health, Mobile OR mHealth OR Telehealth OR eHealth OR Internet OR Web OR Online OR App OR Wearable OR Sensor OR smartphone OR WeChat OR QQ)

3: TS= clinical trial* OR TS=research design OR TS=comparative stud* OR TS=evaluation stud* OR TS=controlled trial*

4: #1 AND #2 AND #3

Database: CNKI

Date searched: 1 Dec 2022

Search Strategy:

--------------------------------------------------------------------------------

1 SU='冠心病' OR SU='冠状动脉粥样硬化性心脏病' OR SU='冠状动脉心脏病'

2  SU='远程康复' OR SU='远程医疗' OR SU='互联网' OR SU='移动医疗' OR SU='微信' OR SU='QQ' OR SU='手机'

3 AB='随机' OR TI='随机' OR SU='随机'

4 1 and 2 and 3

Database: WANG FANG

Date searched: 1 Dec 2022

Search Strategy:

--------------------------------------------------------------------------------

1 主题:( 冠心病) or 主题:(冠状动脉粥样硬化性心脏病) or 主题:( 冠状动脉心脏病)

2 主题:(远程康复) or 主题:(远程医疗) or 主题:( 互联网) or 主题:(移动医疗) or 主题:(微信) or 主题:( QQ) or 主题:(手机)

3 主题:(随机) or 题名:(随机) or 摘要:(随机)

4 1 and 2 and 3
