# Supplementary material for: Longer-Term Effects of Cardiac Telerehabilitation on Patients With Coronary Artery Disease: Systematic Review and Meta-Analysis
Source: JMIR Mhealth Uhealth. 2023 Jul 28;11:e46359. doi: 10.2196/46359 (PMC10422170; doi:10.2196/46359)
Supplement: Multimedia Appendix 2 [file mhealth_v11i1e46359_app2.docx]

**Table S1.** Descriptive characteristics of the 10 studies.

| Author, country | Study design | Follow-up (months) | Population, n; males, n (%) | Age (years), mean (SD) | Diagnosis | Telerehabilitation | Control | Outcomes |
| --- | --- | --- | --- | --- | --- | --- | --- | --- |
| Avila et al [26], Belgium | 3-arm RCT^a^ | 12 | IG^b^: n=26; n=23 (88%)  CG^c^: n=29; n=26 (90%) | IG: 62.2 (7.1)  CG: 62.0 (7.4) | CAD^d^ | Cardiac telerehabilitation group: 3-month training sessions and supervision of the research group for acquaintance with the telemonitoring system (ie, heart rate monitors and data uploading)  Frequency: 6-7 days/week  Intensity: heart rate set at 70%-80% heart rate reserve  Time: at least 150 minutes/week  Type: individualized exercise prescription | Center-based group: 3-month training at the outpatient clinic  Frequency: 3 times/week  Intensity: heart rate set at 70%-80% heart rate reserve  Time: 90 minutes/session (on average 45 minutes at the training heart rate)  Type: endurance training (cycling, running, arm ergometry, rowing, and dynamic calisthenics) followed by relaxation | Primary: CPET^e^ outcomes  Secondary: physical activity, muscle function, cardiovascular risk factors (including BMI, waist and hip circumference, blood pressure, and biochemical analysis of a fasting blood sample), and HRQoL^f^ |
| Batalik et al [27], Czech Republic | 2-arm RCT | 15 | IG: n=23; n=17 (78%)  CG: n=21; n=19 (86%) | IG: 56.1 (6.8)  CG: 57.1 (7.9) | CAD (angina pectoris, MI^g^, with EF^h^>45%, post-PCI^i^ or post-CABG^j^) | Cardiac telerehabilitation group: 3-month training sessions using a heart rate wrist monitor based on telemonitoring  Frequency: 3 times/week  Intensity: heart rate set at 70%-80% heart rate reserve  Time: 60 minutes/session  Type: walking or cycling, aerobic training | Center-based group: monitored by a specialized physiotherapist  Frequency: 3 times/week  Intensity: heart rate set at 70%-80% heart rate reserve  Time: 60 minutes/session  Type: cycling on ergometers and walking on the treadmill | Primary: CPET outcomes  Secondary: anthropometric characteristics (BMI and WC^k^), HRQoL, and number of hospitalizations and deaths |
| Blasco et al [28], Spain | 2-arm RCT | 12 | IG: n=102; n=83 (81.4%)  CG: n=101; n=80 (79.2%) | IG: 60.6 (11.5)  CG: 61 (12.1) | CAD (ACS^l^) | Patients randomized given an automatic sphygmomanometer, a glucose and lipid meter, and a cellular phone for 12-month self-management  Data accessed by a cardiologist through a web interface and recommendations send via the Short Messaging Service  Frequency: unclear  Intensity: unclear  Time: unclear  Type: physically active | Usual-care treatment | Primary: proportion of improvement in blood pressure, BMI, smoking status, LDL-c^m^, and HbA1c^n^  Secondary: quantitative changes in LDL-c, blood pressure, BMI, HbA1c, QoL^o^, and level of anxiety |
| Dorje et al [29], China | 2-arm RCT | 12 | IG: n=156; n=128 (82.1%)  CG: n=156; n=126 (80.8%) | IG: 59.1 (9.4)  CG: 61.9 (8.7) | CAD (MI and unstable or stable angina, post-PCI) | WeChat’s powerful functions, including a cardiovascular health education system, 2-way communication between participants and caregivers, and individualized supervision and medical counseling in 6 months, used  Frequency: 5 times/week  Intensity: 10,000 accumulated steps of walking/day  Time: no strict restrictions  Type: walking | Usual care: usual care provided by community doctors | Primary: 6-minute walk distance test (6MWT)  Secondary: participants’ knowledge and awareness of coronary heart disease, heart rate, blood pressure, CR^p^ needs, plasma glucose, full lipid profile, adherence, smoking status, BMI, waist-to-hip ratio, psychosocial well-being, and QoL |
| Frederix et al [30], Belgium | 2-arm RCT | 24 | IG: n=62; n=52 (84%)  CG: n=64; n=51 (80%) | IG: 61 (9)  CG: 61(8) | CAD | Cardiac telerehabilitation group: 6-week center-based CR and then remaining 6 weeks of center-based CR, in addition to 6 months of cardiac telerehabilitation using both physical activity telemonitoring and dietary/smoking cessation/physical activity telecoaching strategies  Frequency: 3 times/week Intensity: heart rate set at 70%-80% heart rate reserve  Time: minimal 30 minutes/session  Type: exercise protocols based on maximal CPET and calculated BMI:   - high aerobic capacity (VO_2_ peak≥80% predicted): ≥100 steps/minute, 3 times/week, 30 minutes/session - low aerobic capacity (VO_2_ peak<80 % predicted): themselves choose the intensity of exercise sessions - BMI>30 kg/m^2^: 10,000-12,000 steps/day - BMI<30 kg/m^2^: 8000-10,000 steps/day for patients | Center-based group: patients completing a 6-month conventional center-based CR program | Primary: CPET outcomes (peak oxygen uptake [peak VO_2_])  Secondary: self-reported physical activity, cardiovascular risk factor profile, HRQoL, cardiovascular readmission rate, and cost-effectiveness |
| Kraal et al [31], the Netherlands | Two-arm RCT | 12 | IG: n=45; n=40,88.9%)  CG: n=45; n=40,88.9%) | IG: 60.5 (8.8  CG: 57.7 (8.7 | CAD (ACS, MI, UA, post-PCI, or post-CABG) | Cardiac telerehabilitation group: a 3-month training program with wearing the heart rate monitoring device  Frequency: at least two training sessions a week  Intensity: heart rate set at 70% to 85% heart rate reserve which was assessed during the CPET  Time: 45 to 60 minutes  Type: The patient’s preferred training modality in their home environment (e.g. cycling, walking/running, workout at a health club). | Center-based group: a 3-month tailored training program at the outpatient clinic under the direct supervision  Frequency: at least two training sessions a week  Intensity: heart rate set at 70% to 85% heart rate reserve which was assessed during the CPET  Time: 45 to 60 minutes  Type: an individually tailored training program on a treadmill or an electromagnetically braked cycle ergometer. | Primary: physical activity level and physical fitness (CPET outcomes)  Secondary: HRQoL, patient satisfaction, training adherence, and cost-effectiveness |
| Lear et al [32], Canada | 2-arm RCT | 16 | IG: n=38; n=34 (90%)  CG: n=40; n=32 (80%) | IG: 61.7 (10.3)  CG: 58.4 (8.8) | CAD (MI, post-PCI, or post-CABG) | Cardiac telerehabilitation group: 4 months of CR delivered by the internet, called virtual CR program (vCRP), which included online intake forms (medical, risk factor, and lifestyle forms), and scheduled 1-on-1 chat sessions; patients also receiving an off-the-shelf heart rate monitor and a home blood pressure monitor  Frequency: 3 times/week  Intensity: unclear  Time: unclear  Type: education sessions and monthly ask-an-expert group chat sessions | Usual care: patients receiving care from their primary care physician and given simple guidelines for safe exercising and healthy eating habits and a list of internet-based resources | Primary: symptom-limited maximal treadmill exercise test  Secondary: total cholesterol, high-density lipoprotein cholesterol (HDL-c), triglycerides, blood glucose, LDL-c, blood pressure, smoking status, BMI, WC, leisure time physical activity, dietary intake, hospital admissions, and emergency room visits |
| Reid et al [33], Canada | 2-arm RCT | 12 | IG: n=115; n=95 (82.6%)  CG: n=108; n=93 (86.1%) | IG: 56.7 (9.0)  CG: 56.0 (9.0) | CAD (ACS, post-PCI) | Participants accessed the CardioFit website to log their daily activity and complete a series of 5 online tutorials over 6 months and were in email contact with an exercise specialist  Frequency: unclear  Intensity: unclear  Time: unclear  Type: physically active | Usual care: received physical activity guidance from their attending cardiologist and an education booklet | Primary: physical activity  Secondary: self-reported leisure-time physical activity, HRQoL, adverse events |
| Snoek et al [34], the Netherlands | 2-arm RCT | 12 | IG: n=61; n=50 (82%)  CG: n=61; n=50 (82%) | IG: 60.0 (8.4)  CG: 59.0 (10.7) | CAD (ACS, post-PCI, or post-CABG) | Cardiac telerehabilitation group: 6-month training program equipped with a smartphone and Bluetooth-connected heart rate belt  Frequency: 5 days/week  Intensity: moderate intensity defined as intensity above VT_1_ based on CPET  Time: at least 30 minutes/day  Type: patients free to choose type of exercise (ie, walking, cycling) | Usual care: patients receiving a traditional 6-month follow-up program with monthly calls by a research nurse, without advice on physical activity | Primary: CPET outcomes  Secondary: QoL, cardiovascular risk factors, care usage, major adverse cardiovascular events, habitual physical activity, emotional and social functioning |
| Wang et al [35], China | 2-arm RCT | 12 | IG: n=81; n=64 (79%)  CG: n=83; n=72 (87%) | IG: 64 (8.7)  CG: 61.2 (7.1) | CAD (post-CABG) | Intervention group received a 12-month WeChat-based follow-up service—a structured program of cardiac health education, medication reminders, and cardiologist-based follow-up service  Frequency: most days of the week  Intensity: unclear  Time: 30-60 minutes  Type: physically active | Usual care: patients saw their community general practitioners or cardiologists according to self-assessment of cardiovascular health | Primary: medication adherence  Secondary: lifestyle modifications and clinical risk markers (blood pressure, heart rate, BMI, and lipids) |

^a^RCT: randomized controlled trial

^b^IG: intervention group.

^c^CG: control group.

^d^CAD: coronary artery disease.

^e^CPET: cardiopulmonary exercise testing.

^f^HRQoL: health-related quality of life.

^g^MI: myocardial infarction.

^h^EF: ejection fraction.

^i^PCI: percutaneous coronary intervention.

^j^CABG: coronary artery bypass grafting.

^k^WC: waist circumference.

^l^ACS: acute coronary syndrome.

^m^LDL-c: low-density lipoprotein cholesterol.

^n^HbA1c: glycated hemoglobin A1c.

^o^QoL: quality of life.

^p^CR: cardiac rehabilitation.
